# Therapeutic Implications from Sensitivity Analysis of Tumor Angiogenesis Models

**DOI:** 10.1371/journal.pone.0120007

**Published:** 2015-03-18

**Authors:** Jan Poleszczuk, Philip Hahnfeldt, Heiko Enderling

**Affiliations:** 1 Center of Cancer Systems Biology, GeneSys Research Institute, Tufts University School of Medicine, Boston, Massachusetts, United States of America; 2 College of Inter-faculty Individual Studies in Mathematics and Natural Sciences, University of Warsaw, Warsaw, Poland; University of Bari Medical School, ITALY

## Abstract

Anti-angiogenic cancer treatments induce tumor starvation and regression by targeting the tumor vasculature that delivers oxygen and nutrients. Mathematical models prove valuable tools to study the proof-of-concept, efficacy and underlying mechanisms of such treatment approaches. The effects of parameter value uncertainties for two models of tumor development under angiogenic signaling and anti-angiogenic treatment are studied. Data fitting is performed to compare predictions of both models and to obtain nominal parameter values for sensitivity analysis. Sensitivity analysis reveals that the success of different cancer treatments depends on tumor size and tumor intrinsic parameters. In particular, we show that tumors with ample vascular support can be successfully targeted with conventional cytotoxic treatments. On the other hand, tumors with curtailed vascular support are not limited by their growth rate and therefore interruption of neovascularization emerges as the most promising treatment target.

## Introduction

Recruitment of blood vessels through a process called angiogenesis [1] is a hallmark of malignant tumor progression [2–3]. Conventional radiotherapy or chemotherapy are designed to induce gross cell kill in rapidly proliferating populations of cancer cells, with treatment side effects being expected in non-malignant active tissues. Although capable of shrinking the tumor many orders of magnitude, complete tumor eradication is often unachievable [4]. Anti-angiogenic treatment is designed to inhibit the tumor vascular support and thus increasing oxygen tension and inducing tumor cell starvation. This can be achieved either by targeting the neo-vasculature directly or by interfering with pro-angiogenic factors secreted by the tumor [5]. Anti-angiogenic treatment holds the promise of being less patient-specific as the host vasculature is targeted and not the constantly evolving tumor population [6]. Numerous mathematical models have been developed to describe tumor growth, angiogenesis, and response to various treatments approaches at different stages. We set out to study the sensitivity of a tumor to different anti-angiogenic drug treatments at different stages of growth. We compare two different anti-angiogenic agents by local and global sensitivity analysis of parameters describing tumor-vasculature interactions. We utilize an established and a modified mathematical model. The local sensitivity analysis investigates the effect of small variation in a single parameter about its nominal/average value when all other parameters are kept fixed at the estimated values. However, biological systems contain typically substantial variations in almost all parameters values; between patients and even within tumors of a single patient. Hence, it might happen that the model parameter ‘*a*’ is locally the most influential for one patient, while for another patient (with other set of nominal parameters values) it is parameter ‘*b*’. In contrast, a global sensitivity analysis reveals which parameters are the most influential in general by perturbing all parameters simultaneously, assuming patient population heterogeneity and thus parameter values uncertainty.

## Materials and Methods

Hahnfeldt and colleagues proposed a mathematical model of the concept that tumor growth and host blood vessel support is bidirectionally modulated [7]. Tumor volume (*V*) and effective vascular support (*K*) that defines the tumor carrying capacity are time-dependent variables described by a set of coupled ordinary differential equations (ODEs). Tumor growth is assumed to be governed by the Gompertz law:

$$V'=-\varepsilon Vlog(\frac{V}{K}).$$ (C1)

With constant effective vascular support *K = K*_*max*_, initial rapid tumor growth is followed by a slowdown as the tumor volume approaches carrying capacity *K*_*max*_. To account for reciprocal interaction of the tumor with the host vasculature, carrying capacity through vascular support can be described by a variable *K* modulated by the tumor *V*:

$$K'=\overset{\begin{array}{c} \text{spontaneous loss}\\ \text{of functional vasculature} \end{array}}{\overset{︷}{-\mu K}}+\underset{\begin{array}{c} \text{vessels growth}\\ \text{stimulation by the tumor} \end{array}}{\underset{︸}{bV}}-\overset{\begin{array}{c} \text{endogenous inhibition}\\ \text{of vasculature} \end{array}}{\overset{︷}{dKV^{2/3}}},$$ (C2)

where -*μK* represents spontaneous loss of functional vasculature, *bV* represents vessels growth stimulation due to factors secreted by the tumor proportionally to its size, and -*dKV* ^*2/3*^ describes endogenous inhibition of previously generated vasculature due to factors secreted by the tumor proportionally to the tumor surface-to-volume ratio. The specific 2/3 power of *V* in the above equation was derived on the basis of the partial differential equation describing the concentration of angiogenic stimulator/inhibitors, see [7] for further details. Model system Equations (C1) and (C2) describe control tumor growth without treatment. In the literature one can find models with different forms of the stimulation and inhibition terms in Equation (C2) [8–9], but in each case the quantitative behavior of the model remains similar.

The model proposed by Hahnfeldt and colleagues can simulate the effect of anti-angiogenic treatment, and model predictions were successfully compared with experimental data of treatment with TNP-470, Angiostatin and Endostatin [7]. The equation describing the evolution of vascular carrying capacity has originally been developed to include treatment effects:

$$K'=-\mu K+bV-dKV^{2/3}-\overset{\text{anti-angiogenic treatment}}{\overset{︷}{eKg(t)}},$$ (O1)

where *g(t)* is the time dependent concentration of an administered inhibitor. Under the usual pharmacokinetic assumptions [10], *g(t)* is expressed as

$$g(t)=\int_{0}^{t}c(s)exp(-clr(t-s))ds,$$ (C3)

where *c(s)* is the administration rate at time *s* and *clr* is the clearance rate of the considered inhibitor. Let us denote model system (C1) and (O1) as *‘the original model’* describing the response of a tumor to anti-angiogenic treatment. The above model, together with its modifications, has been an object of intensive studies from the point of view of optimal anti-angiogenic treatment scheduling [11–12].

Poleszczuk and coworkers [13] argued that the original model, although successful in predicting the response to therapeutic agents that block the growth of new blood vessels (for example, Angiostatin), might insufficiently describe the effect of anti-angiogenic drugs that act to inhibit angiogenic stimulation. Bevacizumab, a humanized monoclonal antibody that inhibits vascular endothelial growth factor A (VEGF-A), was provided as an example of such agent [13]. For anti-angiogenic agents like Bevacizumab the following modification of the original equation was proposed:

$$K'=-\mu K+\frac{b}{1+\underset{\text{anti-angiogenic treatment}}{\underset{︸}{eg(t)}}}V-dKV^{2/3},$$ (M1)

where, as above, *g(t)* represents the concentration of the administered agent; see [13] for derivation details. Note that in comparison to the original model, treatment does not induce gross reduction of vasculature (c.f., Equation (O1)) but selectively inhibits formation of tumor-stimulated neovasculature. Let us denote model system (C1) and (M1) as *‘the modified model’* describing the response of a tumor to anti-angiogenic treatment.

### Data Fitting

Data fitting of control, original and modified models was performed to experimental data obtained by Fujita and colleagues [14]. The values estimated in the original model [7] were taken as the initial set of parameters for tumor growth without treatment, i.e., the control case. The maximal perturbation value for each parameter was assumed to be equal to 80% of its initial value. For spontaneous death rate *μ*, which was neglected in the original model analysis, we assumed the range of admissible values between 0 and 1 (representing 0%-100%). For treatment associated parameters *D*, *clr* and *e*, we only considered positive values. Data fitting was divided into control case fitting and subsequent treatment model fitting. In both cases, trust region method (incorporated in MATLAB *lsqnonlin* function) for finding the minimal fit error values was utilized. The trust-region-reflective algorithm uses a quadratic approximation for the minimized function (defined by the first two terms of its Taylor approximation) in a neighborhood (trust region) around the current point x to improve the current approximation error [15]. In order to avoid finding only local minima we generated 1000 random initial parameter values for the optimization procedure.

### Sensitivity analysis

We focused on the sensitivity of tumor volume *V* and ignored the sensitivity of effective vascular support *K* (as *V* is directly dependent on *K*). Local sensitivities were obtained by solving the extended system of equations and taking the derivative of the initial vector field with respect to all parameters. To measure global sensitivity we assumed that each parameter is perturbed by a uniformly distributed random variable within the range of ±10% or ± 20% of the initial parameter value. Spearman’s partial rank correlation coefficients were calculated from 1,000 samples generated with a Latin Hypercube Sampling (LHS) algorithm [16]. The scatter plots of obtained samples revealed only monotonic relations between tumor volume and parameters. The sensitivity indices, defined as fraction of total output variance generated by the uncertainty in the respective parameter value, were calculated using the Fourier Amplitude Sensitivity Test (FAST) method [17].

## Results

### Bevacizumab treatment data

**Fig 1:**
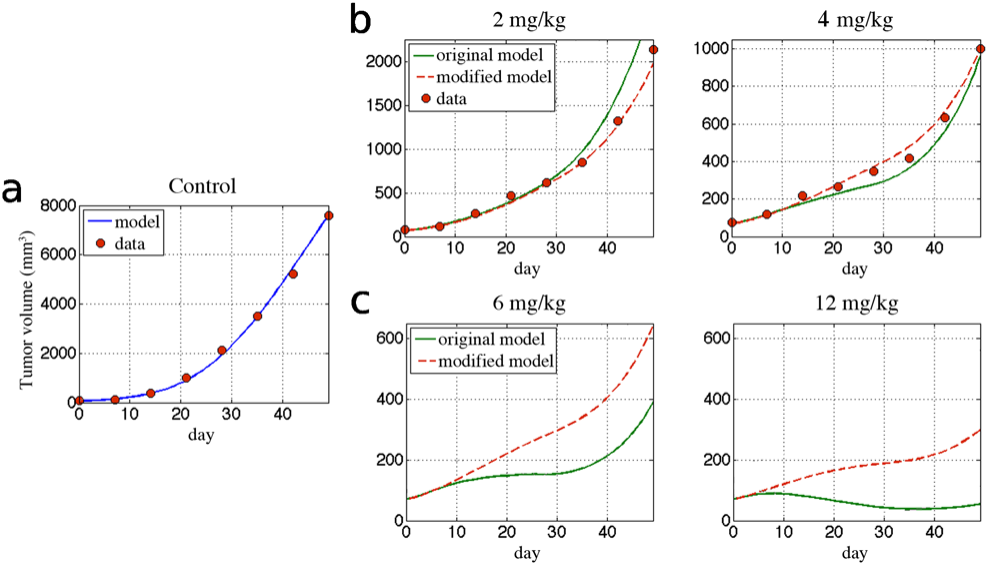
Fitted model curves and predicted tumor response to different doses of bevacizumab. Control data derived from [14] and parameters in Table 1. **(a)** Control data and the fitted model curve using Equations. C1, C2. **(b)** Treatment data together with the solutions to original (Equations. C1, O1) and modified (Equations. C1, M1) models. **(c)** Response of tumor for higher doses of bevacizumab: 6 mg/kg and 12 mg/kg

The impact of Bevacizumab on head and neck squamous cell tumors grown in Female BALB/c nu/nu nude mice was investigated experimentally [14]. Tumor-bearing mice were randomized at mean tumor volume 50–100 mm^3^. Mice were treated with Bevacizumab either 2 mg/kg/day or 4 mg/kg/day on days 1 and 4 of each week for 4 weeks. Both treatment regimes were shown to decelerate growth but are insufficient to prevent tumor expansion (Fig. 1b).

**Table 1:** Estimated tumor growth parameters.

	Description	Control	Original model	Modified model
*e*	drug impact		0.0636	0.4755
*clr*	clearance rate		0.0745	0.0799
*ε*	growth rate	0.0741	0.0741	0.0741
*μ*	loss of vessels	0.0021	0.0021	0.0021
*b*	stimulation	1.3383	1.3383	1.3383
*d*	inhibition	0.002	0.002	0.002
V_0_	initial V	71.2553	71.2553	71.2553
K_0_	initial K	71.6675	71.6675	71.6675
Fit error	average % per point	6.57	2 mg/kg/day: 12.66; 4 mg/kg/day: 10.97	2 mg/kg/day: 7.1; 4 mg/kg/day: 6.26

The kinetic model (Equations. (C1) and (C2)) was applied first to the untreated control tumor data, and the growth parameters *ε*, *μ*, *b*, *d*, and K_0_ (initial value of K) were solved for by performing gradient-based optimization for 1,000 randomly chosen set of initial parameters. Using obtained parameters the data for Bevacizumab (2 or 4 mg/kg) was used to solve for the respective treatment parameters *e* and *clr* in case of the original model (Equations. (C1) and (O1)) and the modified model (Equations. (C1) and (M1)).

Although Bevacizumab was not injected as bolus, for simplicity we assume that *c(s) = D*(δ(*s—t*_*1*_)+ δ(*s—t*_*2*_)+…), where *D* is the administered dose and *t*_*i*_ are the injection days. This assumption may generally lead to decrease in fit quality but should not influence the comparison between considered models. Comparison of the experimental data with fitted curves demonstrates the ability of both models to reproduce the experimental data (Fig. 1a). An excellent control fit was obtained when solving the model (Equations (C1) and (C2)) for parameters *ε*, *μ*, *b* and *d* (Fig. 1a). In contrast to previous assumptions that spontaneous vasculature loss *μ* is negligible [7] we obtained the best fitting curve for *μ>0*, specifically *μ =* 0.0021 (cf. Table 1). This value, however, is relatively small and therefore its role in the model will be addressed in the next sections. A good fit for early response to low doses of Bevacizumab of 2 and 4 mg/kg/day was obtained by both the original and modified model without significant differences in tumor growth curves (Fig. 1b). At the end of the treatment (*t*>30 days), however, the original model fails to correctly approximate tumor growth for the 2 mg/kg/day treatment. For both doses of Bevacizumab the total fit error for the modified model was about two times smaller than for the original model (1.78 for 2 mg/kg/day and 1.75 for 4 mg/kg/day of Bevacizumab), see Table 1 for specific values of fit error. Large differences in treatment predictions by both models are observed for higher doses of bevacizumab (Fig. 1c). For treatment doses of 12 mg/kg/day, the original model predicts a more than three times larger tumor response to treatment than the modified model. Due to lack of data for such a high doses of Bevacizumab, however, we are unable to score the model predictions.

### Treatment free model: sensitivity analysis

**Fig 2:**
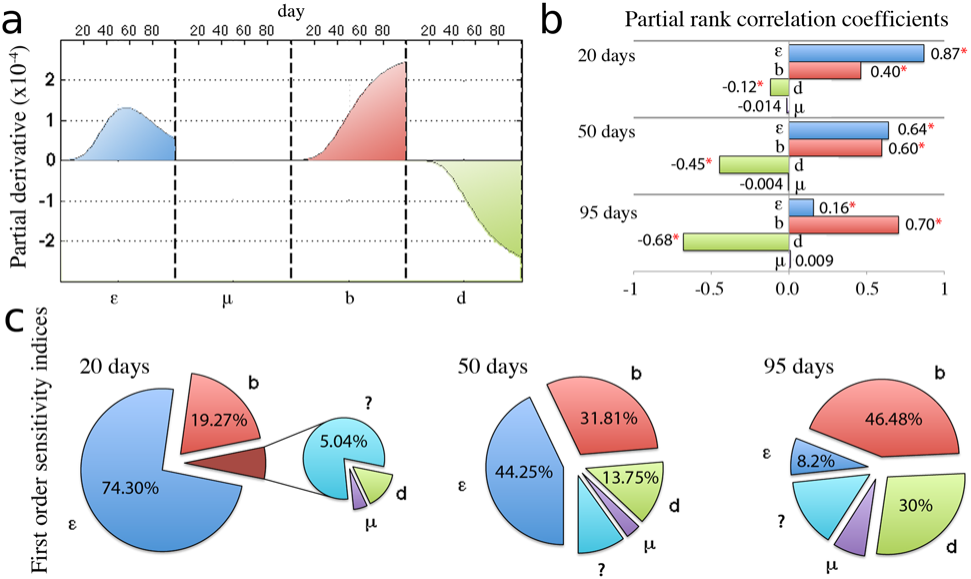
Local and global sensitivity analysis for the control case. Analysis carried with the nominal parameters values presented in Table 1. (a) Time evolution of the local sensitivities, defined as the partial derivatives of the tumor growth curve taken with respect to each parameter and multiplied by the nominal parameter value. (b) Partial rank correlation coefficients between each parameter and the tumor volume after 20, 50 or 95 days from initiation. Coefficients were calculated from 1,000 randomly generated samples under the assumption that each parameter is uniformly varied by no more than 10% of its initial value (* denotes p-value below 0.01). (c) First order sensitivity indices, defined as the fraction of the total variance in tumor volume caused by the variation in each parameter value. Calculations were performed after 20, 50 and 95 days from tumor initiation using the FAST method and under the assumption that parameters are varied uniformly by no more than 10%.

Parameter values obtained in the previous section describe average tumor growth. Each parameter, however, has an intrinsic burden of uncertainty, which is reflected in patient-specific clinical disease courses of tumors of the same organ. In order to obtain the compact form of the considered models, however, many aspects of the tumor angiogenesis process have been incorporated into single parameters, which introduces additional variation in their values. Sensitivity analysis for the treatment-free model described by Equations. (C1) and (C2) demonstrates the influence of the uncertainty in the parameters values (Fig. 2). A basic approach to measuring sensitivity at a fixed time point is to calculate the partial derivatives of systems solution with respect to the parameters [18]. That ‘local’ sensitivity analysis provides direct information on the effect of small variation in a single parameter about its nominal value. Our analysis revealed that variation in the parameter for spontaneous loss of vasculature, *μ* in Equation. (C1), has no significant influence on the tumor size (Fig. 2) and can therefore be neglected in the model as predicted by Hahnfeldt and colleagues [7]. Sensitivity analysis further shows that for early tumor growth up, i.e. *t* < 50 days, there is no significant difference in ‘local’ sensitivity to each parameter (Fig. 2a). In other words, small variation in each single model parameter has similar impact on early tumor growth dynamics. After *t* = 50 days, however, when the tumor has reached an appreciable size, sensitivity to growth rate *ε* begins to decrease while sensitivity to other model parameters continues to increases at similar rates as before. Therefore, tumor growth for larger tumors is not determined by its intrinsic growth rate but increasingly dependent on parameters associated with the angiogenesis process.

Although the performed local analysis provides direct information on the effect of small parameter perturbations about their nominal values, it does not indicate the effect of concurrent, large perturbations in all model parameters. In cancer progression and treatment, many parameters are unknown or only estimated, and may be uncertain by one or more orders of magnitude [18]. In order to reflect this uncertainty, we assume that the value of each parameter is uniformly distributed around the nominal value obtained through data fitting in previous section. We set the range of that distribution to ± 10% of the nominal value. Such amount of uncertainty in each parameter yields up to 30% differences in the tumor volume 100 days after initiation. Fig. 2b and c show the partial rank correlation coefficients and variance decomposition indicative of most influential parameters perturbations. At large tumor sizes local and global sensitivity analysis concur that tumor progression is most sensitive to angiogenesis-associated parameters. Global sensitivity analysis, however, reveals that tumor growth rate *ε* is the major determinant of tumor progression when the tumor is small (Fig. 2c). At the beginning of tumor growth almost 75% percent of variation in tumor volume are due to uncertainty in the tumor growth rate. Similar size dependence is confirmed in the values of partial rank correlation coefficients (Fig. 2b). The initial high correlation of tumor volume with the value of the tumor growth rate decreases in favor of angiogenesis-associated parameters. From the global sensitivity analysis we derive the following therapeutic implications: (*i*) at small tumor sizes it is better to perturb the tumor growth rate by targeting cancer cells directly, and (*ii*) manipulation of the vascular supply by increasing inhibition or decreasing stimulation is the most promising approach when tumors have grown to a substantial size.

### Target angiogenic stimulators or endothelial cells?

**Fig 3:**
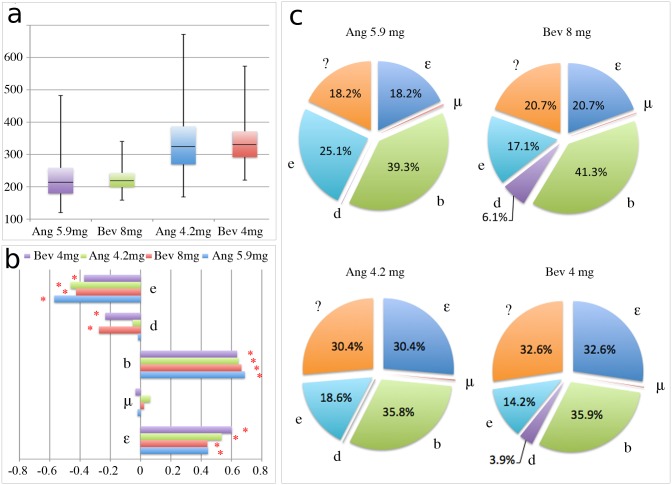
Global sensitivity analysis for Bevacizumab and Angiostatin. Analysis carried for two different dose regimes and with the nominal parameters values presented in Table 1 and Table 2. (**a**) Box plot of tumor volume after 26 days from the initiation calculated from 1,000 samples generated under the assumption that parameters *ε*, *μ*, *b*, *d*, *e* are varied uniformly up to 20%. (**b**) Partial rank correlation coefficients between each parameter and the tumor volume after 26 days from initiation (* denotes p-value below 0.01). (**c**) First order sensitivity indices, defined as the fraction of the total variance in tumor volume caused by the variation in each parameter value. Calculations were performed after 26 days from tumor initiation using the FAST method.

As alluded to above, anti-angiogenic drugs can have different courses of action. One class of drugs interferes with the balance of angiogenesis promoters and inhibitors in favor of inhibition, whereas the other class prevents blood vessel formation regardless of stimulatory signals. We set out to answer the question which anti-angiogenic mechanism is less patient specific and promises more robust treatment results. The original model is used in case of Angiostatin, TNP-470 and Endostatin and the modified model for Bevacizumab. Table 2 shows the pharmacokinetic parameters for Bevacizumab as estimated above (cf. Table 1), as well as TNP-470, Angiostatin and Endostatin as estimated by Hahnfeldt and colleagues through fitting to experimental data [7]. We assume the same treatment protocol as in the previous section with drugs being administered on days 1 and 4 of each week for 4 weeks. Doses for TNP-470, Angiostatin and Endostatin are chosen to give response curves comparable to treatment with Bevacizumab at 4 and 8 mg/kg/day. For sensitivity analysis of the treatment model we only present data for Angiostatin as similar results were obtained for both TNP-470 and Endostatin (data not shown). In order to investigate the robustness of the treatment outcome we revisit the intrinsic uncertainty for parameters *ε*, *μ*, *b*, *d*, *e*. We assume that the variation is uniformly distributed around the nominal value of the parameter and its maximal value is limited to 20%. Fig. 3 shows the global sensitivity analysis revealing the influence of parameter uncertainty on tumor volume 26 days after initiation. For both drug dose regimes a larger variation in tumor volume is observed for Angiostatin than for Bevacizumab. With comparable average tumor volumes at the end of treatment, Bevacizumab yields 34% and 49% lower standard deviation from average tumor size than Angiostatin for both lower and higher drug doses, respectively. The minimal tumor volumes for both drug regimes however, are in favor of Angiostatin, with almost 25% less tumor volume than the smallest tumors obtained with Bevacizumab. On the other hand, the worst possible outcomes (maximal tumor volume) with Bevacizumab were almost 30% smaller than those in the Angiostatin group. The decomposition of the variance revealed no significant differences between both drugs, providing the similar amount of unexplained origin of variation (higher order interactions) (compare Fig. 3c). Differences are only observable in the dependence of tumor volume on uncertainty in parameter *d* describing endogenous vasculature inhibition. Estimated partial rank correlation coefficients show higher correlation of *d* with treatment outcome for Bevacizumab, suggesting that tumors with larger endogenous inhibition of tumor angiogenesis may respond better to Bevacizumab.

**Table 2:** Dosage of different agents giving similar therapeutic effects.

	e (drug impact)	clr (clearance rate)	D (dose)
**TNP-470**	1.3	10.1	13.2; 20.2
**Endostatin**	0.66	1.7	4.1; 5.8
**Angiostatin**	0.15	0.38	4.2; 5.9
**Bevacizumab**	0.4755	0.0799	4; 8

Shown are the exact values of pharmacokinetic parameters (e, clr) and doses (D) for which the original model (TNP-470, Endostatin, Angiostatin) and the modified one (Bevacizumab) give the same tumor volume at the end of treatment. Values of pharmacokinetic parameters, except for bevacizumab, were estimated in [7].

## Discussion

Mathematical models can be utilized to dissect complex mechanisms underlying tumor growth and response to treatment, especially if treatment is not directed at tumor cells but at the environment that modulates tumor growth kinetics. We set out to investigate the sensitivity of parameters in a well-studied mathematical model of tumor growth with reciprocal dependence on its vascular support, and its response to anti-angiogenic treatment [7, 13]. In line with previous assumptions, our analysis confirmed that tumor growth rate as well as tumor-orchestrated angiogenesis promotion and inhibition outweigh spontaneous loss of vasculature in tumor growth dynamics. Local and global sensitivity analysis further revealed that tumor growth is separated into distinct phases with different dependence on underlying mechanisms. Intuitively, when a tumor is initiated its progression is predominantly determined by the intrinsic growth rate, that is, ratio of cell proliferation to cell death. As the tumor grows and exhausts its vascular support the growth rate becomes insignificant and tumor progression is increasingly dependent on the interplay of angiogenesis promoting and inhibiting mechanisms. These findings offer valuable insights into tumor size-dependent treatment design. Small tumors as well as tumors progressing with ample vascular support might be best targeted by direct induction of cell kill. Tumors with curtailed vascular support are not dependent on their growth rate, and induction of cell kill will only have minimal effects. We showed that for such tumors interference with the neovascularization is the most promising treatment target. These model predictions will need to be confirmed in carefully designed animal experiments that allow for quantification of the degree of tumor vascularization and vascularization-dependent response to cytotoxic and anti-angiogenic treatments as well as their combinations.

A number of anti-angiogenic treatments have recently been approved for both single treatment and in combination with other therapeutic agents and many more are in various stages of clinical trials. The Hahnfeldt model has successfully predicted tumor response to anti-angiogenic treatment with TNP-470, Angiostatin and Endostatin [7]. A modification of that model put forward by Poleszczuk and colleagues extended its applicability to treatment with Bevacizumab [13]. We normalized treatment protocols in both models to achieve comparable tumor sizes after treatment and analyzed the sensitivity of both models to the underlying parameters. With equal perturbation of parameters in both models representative of patient variability we showed that the average tumor sizes after treatment with both Angiostatin and Bevacizumab are similar. The deviation from average response, however, was significantly larger for Angiostatin, indicating that the course of action with Bevacizumab is less patient specific and thus wider applicable. The most favorable outcome, however, was observed for treatment with Angiostatin, with final tumor size being more than 30% smaller than the best sample from the Bevacizumab group. On the other hand, the least successful treatment outcome featured also a significantly larger tumor in the Angiostatin group compared to Bevacizumab. No significant differences in parameter sensitivity were found between both drugs, providing similar amount of unexplained origin of variation in tumor response. These results visualize that higher order interactions between tumor, vasculature and anti-angiogenic agents are at play that are yet to be fully deciphered.

In conclusion, we have demonstrated that simple mathematical models with a small number of experimentally validated parameters can reliably reproduce and predict tumor growth and treatment response data. Thorough analysis of parameter uncertainty yields invaluable insights into mechanisms driving growth kinetics and response of tumors. Our study encourages the measure of tumor vasculature as a surrogate for tumor carrying capacity as a biomarker, which may ultimately lead to better-informed patient-specific synergizing of cytotoxic and anti-angiogenic treatment. These findings from our analysis augment the understanding of cause-action relation of tumor kinetics and aim to drive future experiments and clinical validation towards improved understanding and ultimately patient prognosis.
